# Control of Phage Bxb1 Excision by a Novel Recombination Directionality Factor

**DOI:** 10.1371/journal.pbio.0040186

**Published:** 2006-05-30

**Authors:** Pallavi Ghosh, Laura R Wasil, Graham F Hatfull

**Affiliations:** 1Pittsburgh Bacteriophage Institute and Department of Biological Sciences, University of Pittsburgh, Pittsburgh, Pennsylvania, United States of America; Centre de Génétique MoléculaireFrance

## Abstract

Mycobacteriophage Bxb1 integrates its DNA at the *attB* site of the Mycobacterium smegmatis genome using the viral *attP* site and a phage-encoded integrase generating the recombinant junctions *attL* and *attR*. The Bxb1 integrase is a member of the serine recombinase family of site-specific recombination proteins and utilizes small (<50 base pair) substrates for recombination, promoting strand exchange without the necessity for complex higher order macromolecular architectures. To elucidate the regulatory mechanism for the integration and excision reactions, we have identified a Bxb1-encoded recombination directionality factor (RDF), the product of gene *47*. Bxb1 gp47 is an unusual RDF in that it is relatively large (˜28 kDa), unrelated to all other RDFs, and presumably performs dual functions since it is well conserved in mycobacteriophages that utilize unrelated integration systems. Furthermore, unlike other RDFs, Bxb1 gp47 does not bind DNA and functions solely through direct interaction with integrase–DNA complexes. The nature and consequences of this interaction depend on the specific DNA substrate to which integrase is bound, generating electrophoretically stable tertiary complexes with either *attB* or *attP* that are unable to undergo integrative recombination, and weakly bound, electrophoretically unstable complexes with either *attL* or *attR* that gain full potential for excisive recombination.

## Abstract 2

The authors identify a protein that employs a new mechanism to regulate the directionality of integration of a mycobacteriophage integrase into its host genome.

## Introduction

Integration of bacteriophage genomes into the chromosomes of their hosts is typically accomplished by integrase-mediated site-specific recombination between *attP* and *attB* sites located in the phage and bacterial genomes respectively [ 1, 2]. Integration generates the junction sites— *attL* and *attR*—that flank the integrated prophage and are used as substrates for excision of the genome during prophage induction. The phage-encoded integrase protein (gpInt) catalyzes both integration and excision, but the reactions are highly directional, with the selection of integrative (using *attP* and *attB*) or excisive (using *attL* and *attR*) recombination determined by accessory proteins called recombination directionality factors (RDFs) [ 3].

Phage integrases can be grouped into two main classes, the tyrosine and serine recombinase families. Perhaps the best-studied member of the tyrosine integrase group is that encoded by phage lambda, in which the DNA sites and protein components are well characterized and structural information on proteins and protein–DNA complexes is now available [ 2, 4– 6]. The basis for directional control of lambda site-specific recombination is well defined, with the RDF (lambda Excise, Xis) [ 7] functioning as a DNA-binding and DNA-bending protein [ 8, 9] whose action specifically alters the formation of higher-order macromolecular architectures [ 10]. These structures are generated by the binding of Int to a relatively large (234 base pair [bp]) *attP* site [ 11] that contains both core-type (C and C′) and arm-type Int binding sites (P1, P2, P′1, P′2, and P′3) [ 12, 13] together with the accessory proteins IHF (binding sites HI, H2, and H3) and Xis (binding sites X1 and X2) [ 14]. Both IHF and Xis introduce substantial DNA bends at their sites and influence the ability of Int to form intra- or inter-molecular bridges through bivalent binding of Int to core- and arm-type sites [ 15]. When Xis is absent, the *attP*/Int/IHF intasome is competent to capture naked *attB* DNA, and strand exchange occurs to generate *attL*/ *attR* product complexes that are unable to undergo excisive recombination [ 16, 17]. When Xis is present, its binding to the P arm of *attP* precludes formation of an *attP* intasome that is proficient for integration (resulting in strong inhibition of the reaction) [ 10]; binding of Xis to *attR* promotes the formation of an *attR* intasome that is competent to synapse productively with an *attL* intasome enabling excisive recombination [ 18], and integrase and excise make close protein contacts [ 19– 21]. Interestingly, several RDFs have functions outside of recombination, including the Cox proteins of phages P2 and HP1, and AlpA of the Escherichia coli genomic island CP4–57 that act as transcriptional regulators [ 22– 24].

The mechanism and control of site-specific recombination events mediated by serine integrases is much less well understood than for the tyrosine integrases. This group of proteins is still relatively small (˜30) compared to the tyrosine integrases (>500) and was only discovered in the mid 1990s [ 25], more than 30 years after the Campbell model for lambda integration [ 26]. The serine integrases are relatively large (500–900 amino acids [aa]) and diverse, but all contain an N-terminal segment (˜150 aa) that is shared with the catalytic domains of transposon resolvases and DNA invertases of the serine recombinase class [ 27– 29]. In vitro integration systems have been established for Streptomyces phage φC31 [ 30, 31], Lactococcal phage TP901–1 [ 32, 33], Mycobacteriophage Bxb1 [ 34] and the Mycobacterium tuberculosis prophage-like element, φRv1 [ 35, 36]. In each example, the *attP* and *attB* sites are small (<50 bp) and likely contain only sufficient sequence information for the binding of two protomers of gpInt [ 36, 37]. The two sites typically contain partial dyad symmetry flanking a central dinucleotide with gpInt cleaving such as to generate two-base 3′ extensions [ 37]. All mechanistic aspects of strand exchange—including covalent linkage of gpInt to the cleaved DNA via a phosphoserine bond—appear to be similar to that of the well-studied resolvase and DNA invertase systems [ 27, 28, 37].

Mycobacteriophage Bxb1 is a temperate phage of M. smegmatis and integrates its genome at an *attB* site located within the 3′ end of the *groEL1* gene [ 34], leading to a defect in biofilm formation of Bxb1 lysogens [ 38]. Integration is catalyzed by a 500-aa serine integrase that is composed of two domains, an N-terminal 150-aa catalytic domain, and a 350-aa C-terminal domain that confers DNA binding [ 39]. The minimally functional recombination sites are 48 bp *(attP)* and 38 bp *(attB)* in length [ 37] and are substantially different at the sequence level, although both contain interrupted inverted repeats flanking a 5′-GT central dinucleotide. In vitro, integrative recombination is both efficient and directional, converting greater than 95% substrate DNAs to the *attL* and *attR* products [ 37], and unlike the resolvase/DNA invertase systems, there is no requirement for DNA supercoiling; gpInt is the only protein component, and no accessory proteins are involved [ 34, 37, 39]. Curiously, synapsis is permissive with respect to site-alignment, with *attP* and *attB* aligning equally efficiently in parallel and antiparallel configurations, both of which proceed through strand cleavage and rotation. However, the non-palindromic nature of the central 5′-GT dinucleotide results in a mismatch in the recombinant configuration when the sites are misaligned, and the intermediates are resolved by a second strand rotation and religation in the substrate arrangement [ 37].

It is unclear how the directionality of serine integrase–mediated site-specific recombination is regulated, since these systems typically do not contain accessory integrase or cofactor DNA-binding sites common in the tyrosine integrase systems, nor are they controlled by DNA topology as in the resolvase/DNA invertase members of the serine recombinase family. In two examples of serine integrases—φRv1 [ 35, 36] and TP901–1 [ 32]—RDFs have been described, both of which are small and basic, and have limited sequence similarity to previously described RDFs that act in tyrosine integrase systems. The mechanism of action of these cofactors in unknown, although the φRv1 system uses small simple substrates for both integration and excision, suggesting that directionality does not involve the remodeling of higher order macromolecular architectures (L. Bibb and G. F. Hatfull, unpublished data).

Here, we report the identification and characterization of an RDF that regulates the directionality of recombination by the Bxb1 integrase. This RDF (Bxb1 gp47) is unusual in that it is relatively large (255 aa), unrelated to all other RDFs, and likely has functions outside of recombination, since close relatives are present in other mycobacteriophages that utilize tyrosine integrase systems and for which all the recombination functions are defined. Bxb1 gp47 is a required accessory protein for excisive recombination both in vitro and in vivo, and is a potent inhibitor of integrative recombination in vitro. However, it does not have DNA-binding activity, only small simple excision substrates are required, and it does not function through remodeling of higher order structures. In contrast to other RDFs, Bxb1 gp47 appears to act via direct protein interactions with gpInt, but in a conformation-dependent manner that is determined by the specific DNA substrate bound by gpInt. Inhibition of integration is facilitated by stable association of gp47 with *attB*–gpInt or *attP*–gpInt complexes to prevent further participation in recombination, whereas excision is strongly stimulated without stable association to *attL*–gpInt or *attR*-gpInt complexes and presumably involves transient but productive associations.

## Results/Discussion

### Isolation of a Bxb1 DNA Segment Promoting Excisive Recombination

**Figure 1:**
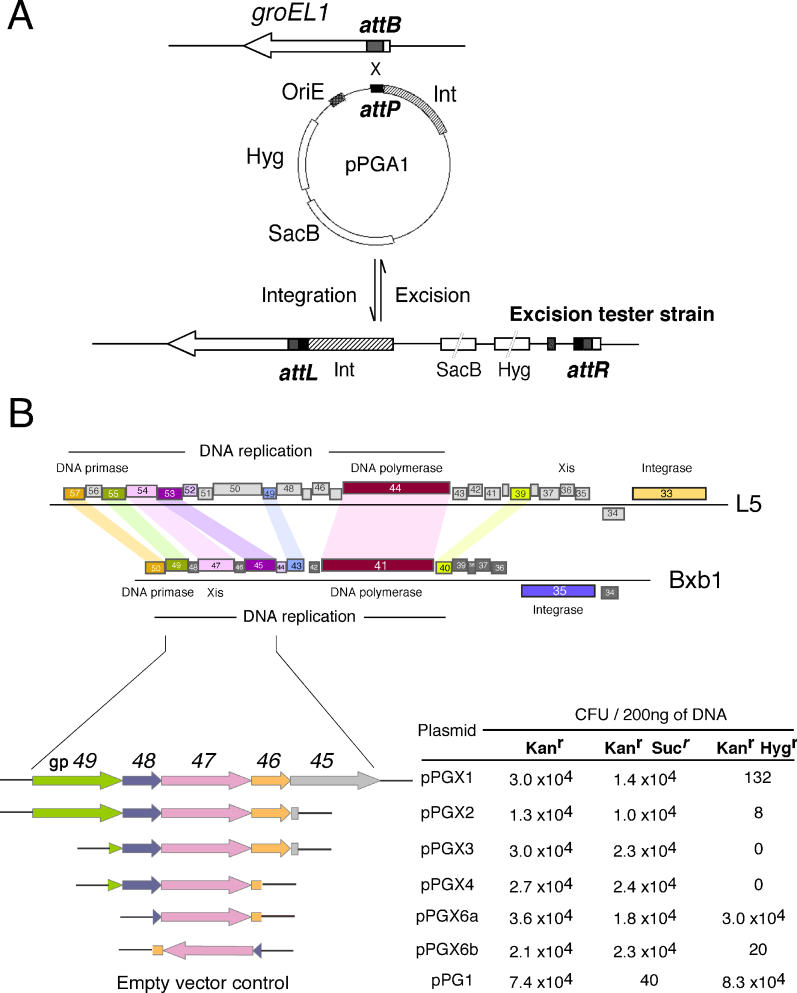
Identification of the Bxb1 RDF (A) The hygromycin-resistance cassette, sucrose-sensitivity cassette *(sacB),* and a portion of the Bxb1 DNA containing *attP* and *int* were cloned into an integrative plasmid containing the plasmid ColE1 origin of E. coli (OriE) *,* and the resultant plasmid pPGA1 was transformed into M. smegmatis mc ^2^155. Expression of *int* drives integration of the plasmid into the host *attB* site leading to the formation of *attL* and *attR* sites flanking the *hyg* and *sacB* cassettes. The resultant strain, designated as the excision tester strain, is resistant to hygromycin and sensitive to the presence of sucrose. An excisive recombination event between *attL* and *attR* results in the removal (and subsequent loss) of the intervening DNA containing *int, hyg,* and *sacB;* the strain consequently becomes *hyg ^S^* and *suc ^R^* and can thereby be monitored by the appearance of colonies in the presence of sucrose. (B) A segment of the Bxb1 genome containing genes involved in integration and DNA replication as well as the corresponding portion of the phage L5 genome is shown in the upper part; related genes are colored accordingly. The lower part of the figure shows the region of Bxb1 DNA present in plasmids exhibiting excision activity. Following isolation of plasmid pPGX1—which is active in promoting sucrose resistance in the excision tester strain shown in (A)—plasmid derivatives containing deletions from both ends of pPGX1 were constructed, introduced into the excision tester strain, and scored for the appearance of *suc ^R^* colonies. CFU, colony-forming units

Using bioinformatic analysis of the Bxb1 genomic sequence, we have not been able to identify candidate proteins showing sequence similarity to previously identified RDFs; this is however not surprising as excises comprise a diverse group of proteins with little sequence conservation. In order to identify and characterize the Bxb1 recombination directionality factor a positive selection assay using a strain reflecting the organization of genes in a Bxb1 lysogen with respect to *attL, attR,* and *int* was established. A portion of the Bxb1 DNA containing *attP* and *int* was cloned into an integrative plasmid containing the hygromycin resistance cassette and the sucrose sensitivity cassette *(sacB),* and the resultant plasmid pPGA1 was electroporated into M. smegmatis mc ^2^155. As described previously [ 34], *int* expression promotes integration of plasmid DNA into the host *attB* site leading to the formation of *attL* and *attR* flanking the *hyg ^R^* and *sacB* cassettes ( Figure 1A). The resulting strain, designated as the excision tester strain, is resistant to hygromycin and sensitive to sucrose. An excisive recombination event between *attL* and *attR* should result in removal (and subsequent loss) of the intervening DNA containing *int, hyg,* and *sacB;* the strain thus becomes *Hyg ^S^* and *Suc ^R^* and is able to grow in the presence of sucrose.

A genomic DNA library of phage Bxb1 was constructed in a vector (pPG1) containing *oriE, oriM,* and the *kan ^R^* marker. The frequency of excision of pPGA1 in the absence of phage sequences was determined by transforming the empty vector (pPG1) into the tester strain; the frequency of *suc ^R^* colonies was less than 0.05%, indicating that the integrated DNA is stably maintained ( Figure 1B). Transformation of a Bxb1 DNA library into the tester strain yielded approximately 10 ^4^ *Kan ^R^* transformants of which 45 were resistant to sucrose. Sequencing the junctions of plasmid DNA recovered from 14 *suc ^R^* colonies revealed the presence of Bxb1 DNA spanning the coordinates 34,440–37,520 bp; the region 35,845–36,800bp was common to all 14 clones and included the previously annotated genes *46* and *47* ( Figure S1). Derivatives of one clone, pPGX1, containing genes *49*– *45,* were constructed with truncations from each end and tested for the ability to generate *suc ^R^* colonies ( Figure 1B). The smallest segment with excision properties was contained within plasmid pPGX6 and spans 35,976–36,811 bp of the Bxb1 genome ( Figure 1B). Efficient excision is observed when this segment is cloned in one orientation (pPGX6b) but not in the other (pPGX6a), presumably reflecting differences in gene expression levels.

**Figure 2:**
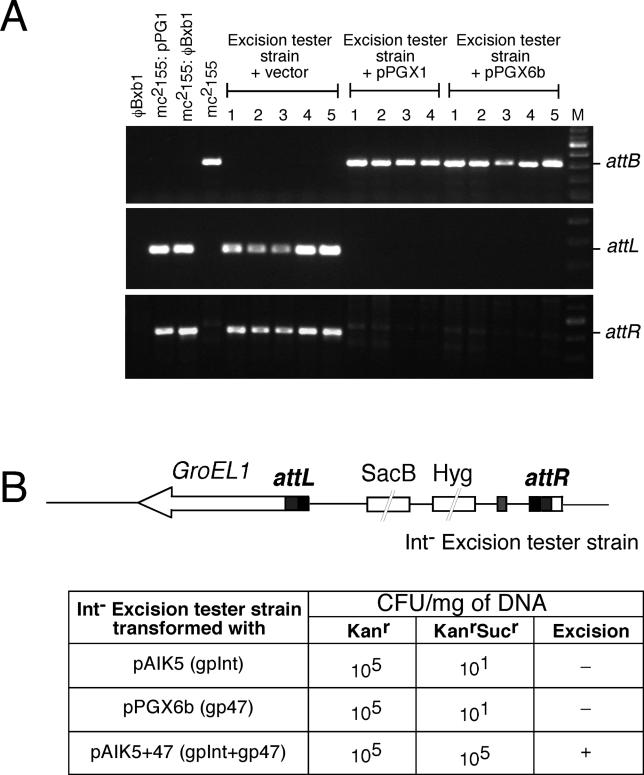
Confirmation of Site-Specific Recombination and Bxb1 Integrase Dependence (A) DNA from five sucrose-resistant transformants of the excision tester strain transformed with either pPG1 (empty vector), pPGX1, or pPGX6b was examined by PCR for the presence of *attB, attL,* and *attR*. Transformation with pPGX1 and pPGX6b leads to the presence of a product amplified with *attB*-specific primers; no product is observed using primers that amplify *attL* and *attR.* DNA from a Bxb1 lysogen and from clones transformed with the empty vector that were used as controls show a product corresponding to *attL* and *attR,* whereas DNA from wild-type *mc ^2^155* shows the presence of a product corresponding to *attB*. (B) An *int ^−^* excision tester strain was created (as in Figure 1A) using transient expression of gpInt. The int ^−^ tester strain was then transformed with pAIK5 (gpInt alone), pX6b (gp47 alone), or pAIK5+47 (gpInt + gp47), and the frequency of suc ^R^ colonies (and therefore excision) determined. CFU, colony-forming units

To determine if sucrose resistance derives from site-specific excisive recombination, DNA from *kan ^R^ suc ^R^* colonies transformed with pPGX1 and pPGX6b, as well as from colonies transformed with vector pPG1, was examined by PCR for the presence of *attB, attL,* and *attR* (excisive recombination should restore *attB* and lead to a loss of the *attL* and *attR* sites). All clones transformed with the vector alone contain products corresponding to *attL* and *attR,* as does a Bxb1 lysogen and the excision tester strain ( Figure 2A); clones transformed with pPGX1 and pPGX6b have lost *attL* and *attR,* but show a product corresponding to *attB* ( Figure 2A). The identity of the PCR products was confirmed by sequencing (unpublished data). These data verify that sucrose resistance is a consequence of site-specific recombination between *attL* and *attR* and that excision is dependent on a fragment of Bxb1 DNA.

To determine the requirement of Bxb1 gpInt in excision, a derivative of the tester strain lacking *int* was created; the integrative plasmid employed here contains only *attP* and was inserted into the *attB* site of M. smegmatis mc ^2^155 by a transient expression of gpInt. The *int ^−^* tester strain was then transformed with pAIK5 (gpInt alone), pPGX6b, or pPGX6bInt (pPGX6b with gpInt). Excisive recombination was found to be dependent on the presence of *int* ( Figure 2B), demonstrating that both gpInt and a putative RDF are required for excisive recombination of Bxb1.

### Bxb1 gp47 Is an RDF

**Figure 3:**
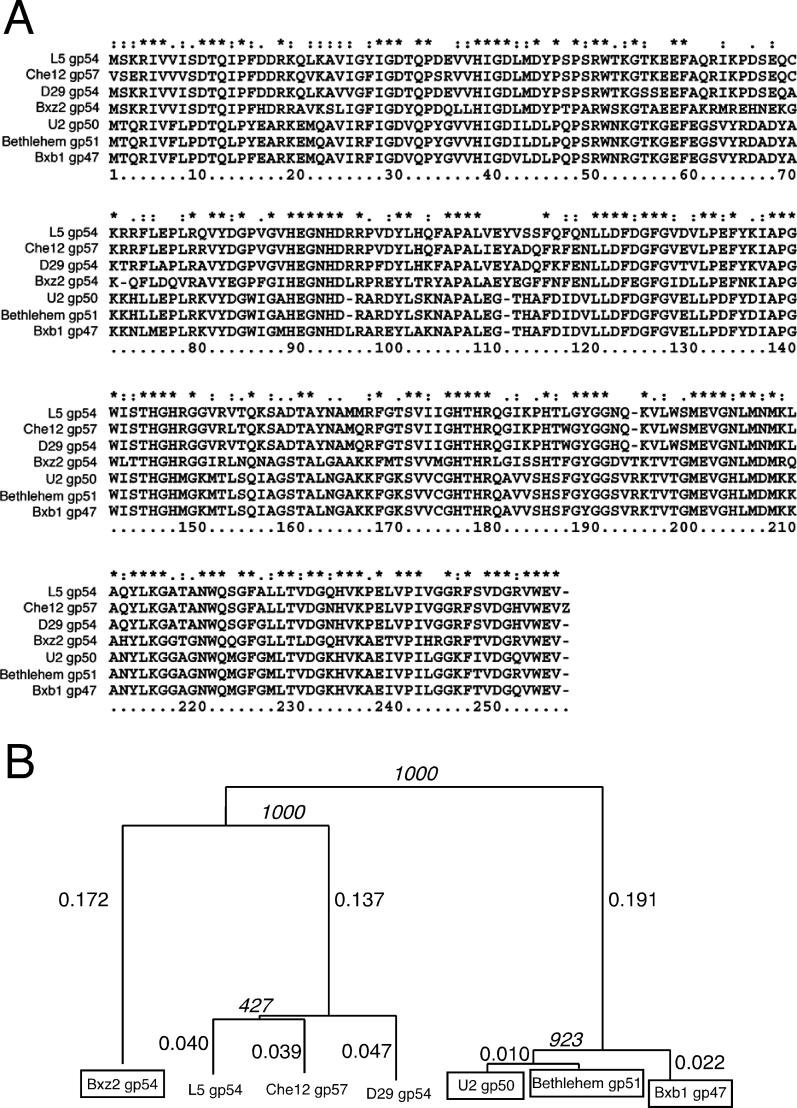
Sequence Alignment of Bxb1 gp47 and Its Homologs (A) A BLAST search identified proteins of mycobacteriophages U2 (gp50), Bethlehem (gp51), L5 (gp54), D29 (gp54), Che12 (gp57), and Bxz2 (gp54) with similarity to Bxb1 gp47. Multiple sequence alignment of these proteins was performed using ClustalW (identical residues indicated by an asterisk [*]) and the phylogenetic relationship (B) represented using NJPLOT; branch lengths and bootstrap (italicized) values are indicated. The proteins encoded by phages that encode a serine integrase (Bxz2, U2, Bethlehem, and Bxb1) are boxed; all others (L5, D29, and Che12) encode a tyrosine integrase.

The Bxb1 sequence in pPGX6 contains no open reading frames (ORFs) encoding proteins with sequence similarity to previously identified RDFs, although this is perhaps not surprising given the high diversity of the RDFs [ 3]. The only previously annotated ORF in this region is gp47, which not only has no similarity to other RDFs, but is closely related to predicted proteins of unknown function encoded by mycobacteriophages Che12 (gp57), U2 (gp50), Bethlehem (gp51), Bxz2 (gp54), L5 (gp54), and D29 (gp54) [ 40– 42] ( Figure 3A). However, only phages U2, Bethlehem, and Bxz2 are predicted to encode serine integrases, whereas L5, D29, and Che12 utilize tyrosine integrases. Furthermore, all of the phage-encoded requirements for L5 recombination have been established [ 43] and do not include L5 gp54, the homolog of Bxb1 gp47. Presumably Bxb1 gp47 and its related proteins play a role other than in site-specific recombination; given their location between DNA polymerase and DNA primase genes ( Figure 1B), we speculate that they may also play a role in DNA replication. Phylogenetic analysis of the Bxb1 gp47-related proteins suggests that acquisition of a role in recombination was not a recent event, since the Bxz2 member of this family is more closely related to the homologs found in the tyrosine integrase phages L5, D29, and Che12 than to other phages encoding serine integrases ( Figure 3B). We note that although a dual function is unusual, there are several such examples among accessory proteins for site-specific recombination, including E. coli PepA, ArgR, and ArcA [ 44], and phages P2 and HP1 Cox proteins [ 23, 24].

**Figure 4:**
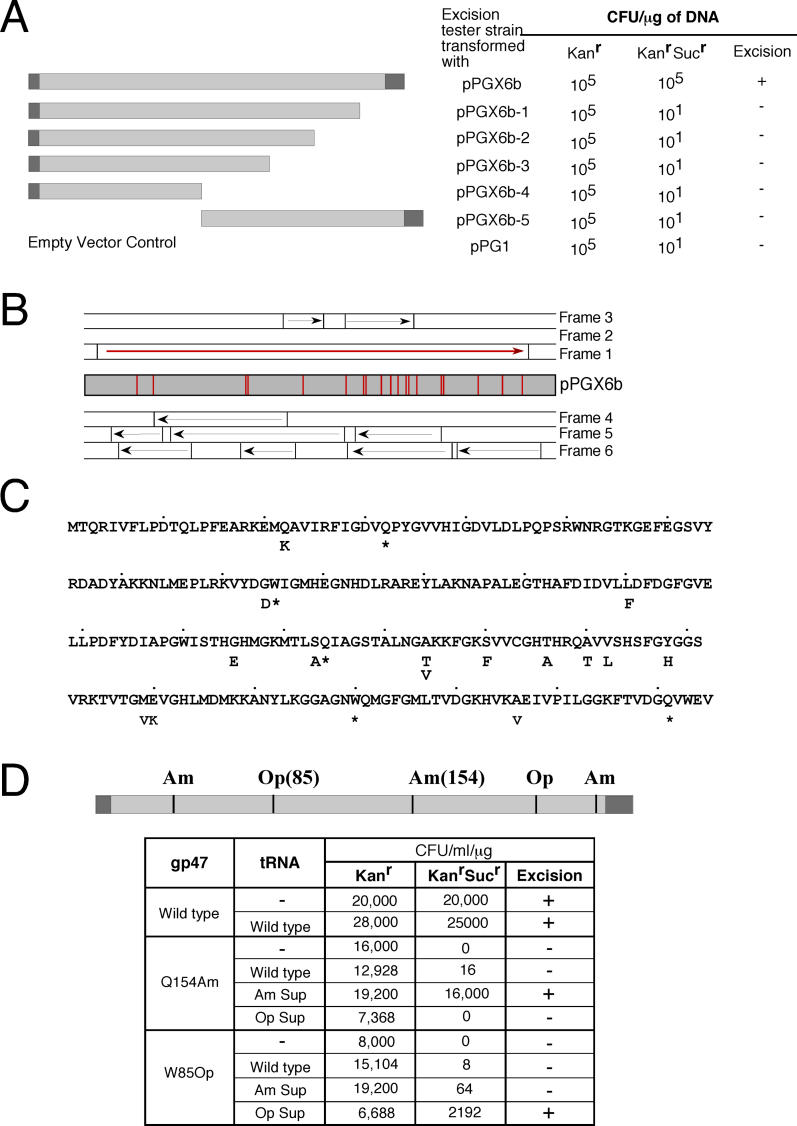
Confirmation of Bxb1 gp47 as the Phage RDF (A) The solid bar shows the portion of Bxb1 DNA from 35,976–36,811 bp contained within pPGX6b with the regions flanking gene *47* in dark grey. Truncation derivatives of pPGX6b were constructed as shown and tested for excision activity as in Figure 1. The number of sucR colonies obtained upon transformation of the excision tester strain with each of the derivatives is shown. (B) Plasmid pPGX6b was randomly mutagenized by passage through a mutator strain, and excision defective mutants were selected ( Figure 1). The positions of 20 excision-defective mutants are shown as solid vertical lines (red), all of which lie within gene *47*. Arrows indicate ORFs in all six reading frames of pPGX6b, with Bxb1 gene *47* shown in red. (C) The amino acid changes in Bxb1 gp47 corresponding to each of the 15 base changes in pPGX6b are shown; the five nonsense substitutions are marked with an asterisk (*). (D) The locations of five nonsense mutations inactive in excision are shown using solid vertical lines. An excision tester strain previously transformed with plasmids expressing gp47 (wild type) or either of the two nonsense mutations (Q154Am or W85Op) was transformed with plasmids expressing nonsense suppressors generated by modification of tRNAs encoded by mycobacteriophage L5 [ 41] (as indicated) and scored for the appearance of sucR colonies; the numbers of sucR colonies are shown. CFU, colony-forming units

The observation that Bxb1 gp47 is unrelated to other RDFs (including the two previously described serine integrases for phages TP901 and φRV1), and that it is larger than all previously characterized RDFs (28 kDa, 255 aa) raises the question as to whether gp47 provides the excision activity, or whether excision function is conferred by the products of one or more other ORFs within this region. We note for example the involvement of three small ORFs in a contiguous DNA segment of the *Bacteroides* conjugative transposon CTnDOT that are required for excisive recombination [ 45]. To address this question, we have used three approaches. First, the 835-bp segment of Bxb1 DNA within plasmid pPGX6b was truncated from both ends, and all truncations are inactive in excision ( Figure 4A). Second, pPGX6b was randomly mutagenized by passage through the mutator strain XL1Red, and excision defective mutants were selected on media containing hygromycin (see Figure 1). DNA from 20 mutants was isolated and sequenced, all of which contain mutations within frame 1 encoding gp47 ( Figure 4B and 4C); furthermore, every base change results in an amino acid (or nonsense) substitution in frame 1 (corresponding to gene *47*) whereas base changes in other frames contain those that are conservative and do not generate amino acid substitutions ( Table S1). Of the 20 mutations, five generate nonsense codons in gene *47,* ten are changes in the first codon position, and the remaining five have changes in the second position; all 15 missense mutations result in an amino acid change in gp47.

Finally, to exclude the possibility of multiple ORFs encoding excision activity, two of the five nonsense mutations ( Figure 4C) in the coding frame of gene *47* were examined by suppressor analysis. The presence of a cognate opal or amber tRNA suppressor (modified tRNAs of mycobacteriophage L5 [C. Peebles and G. F. Hatfull, unpublished data]) was able to restore excision activity of gp47W85Op and gp47Q154Am, respectively; no excision was observed by the mutant gp47 when either a wild-type tRNA or a non-cognate suppressor tRNA was used ( Figure 4D). Taken together, these results unequivocally demonstrate that gp47 is the protein responsible for stimulating excisive recombination.

### Overexpression of gp47 and Establishment of an In Vitro Excisive Recombination Assay

**Figure 5:**
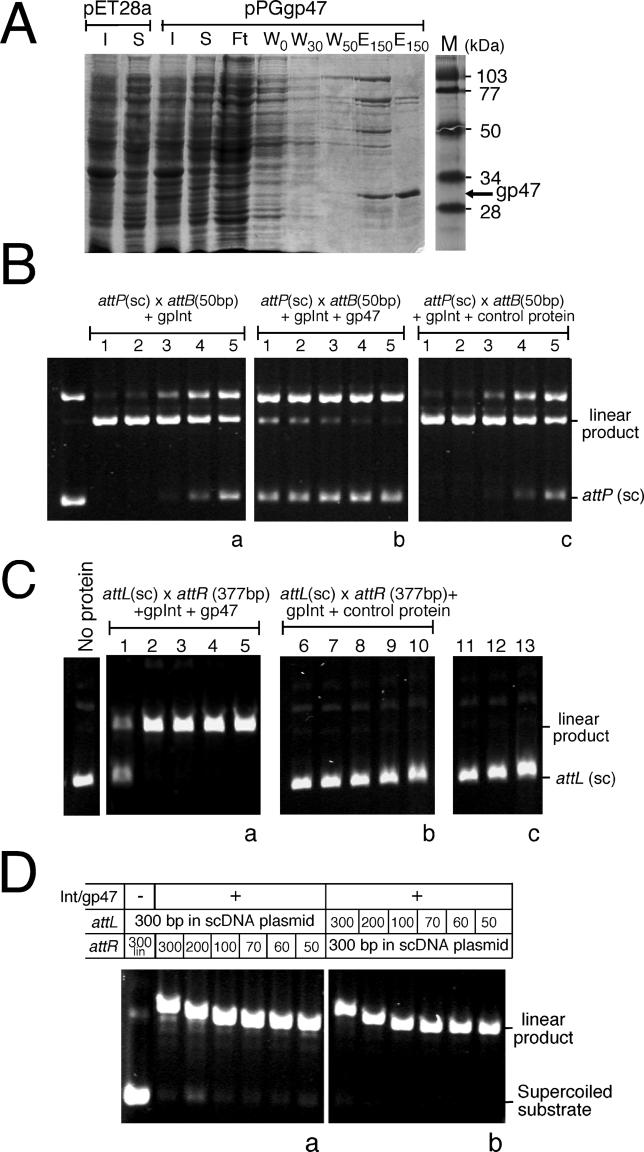
In Vitro Excisive Recombination Using gp47 (A) E. coli BL21(DE3)pLysS transformed with pET28a and pPGgp47 were grown to an A _600_ of 0.6 at 30 °C and induced for an additional 4 h at 22 °C with 0.6 mM IPTG. The cells were lysed in lysis buffer (see Materials and Methods) and partially purified by passage through a Ni-NTA column followed by elution with 150 mM imidazole; I, S, Ft, W, and E represent the insoluble fraction, soluble fraction, flow-through from the Ni-NTA column, washes with the indicated concentration of imidazole, and 150 mM elution from the Ni-NTA column, respectively. The induced cells of pPGgp47 show the presence of an approximately 32-kDa protein (as indicated) that is absent from the pET28a expression lanes and is abundant in the insoluble fraction. Molecular weight markers are shown in lane M and their corresponding sizes indicated. (B) Integrative recombination was performed as described previously using a supercoiled *attP* substrate, a linear 50-bp *attB* DNA, and increasing concentrations of gpInt [ 34]: panel a, in the absence of any additional protein; panel b, in the presence of partially purified gp47; panel c, with addition of a control extract. Lanes 1–5 contain 0.36, 0.18, 0.09, 0.045, and 0.0225 μM of gpInt respectively. Panels b and c contain 1.78 μM of gp47 and an equivalent amount of the control extract respectively, in addition to gpInt. The positions of the supercoiled substrate and the linear recombinant product are indicated. The small (50 bp) linear *attB* substrate migrates fast and is not shown. (C) Excisive recombination was carried out in recombination buffer (see Materials and Methods) using a 367-bp *attL* in a supercoiled plasmid and a 377-bp linear *attR* partner DNA. Lanes 1–5 of panel a contain increasing concentrations of gp47 (0.89, 1.78, 2.67, 3.56, and 5.34 μM), lanes 7–11 of panel b contain an equivalent amount of the control protein. Control reactions lacking either the partner *attR* DNA (lane12), gp47 (lane 13), or gpInt (lane14) are shown in panel c. The positions of the supercoiled *attL* substrate and the linear recombinant product are indicated. (D) Excisive recombination reactions with varying sizes of linear DNA substrates show that only small substrate sites are required. Panel a shows recombination between a supercoiled plasmid containing a 367-bp *attL* and varying sizes of linear *attR* partner DNA as indicated. Panel b shows recombination between a supercoiled plasmid containing a 377-bp *attR* and varying sizes of linear *attL* partner DNAs. The positions of supercoiled substrates and linear recombinant products are indicated.

Bxb1 gene *47* was cloned in the pET28 expression system such as to generate a fusion protein with an N-terminal His tag. Cells transformed with the resultant plasmid (pPGgp47) were grown to A _600_ of approximately 0.4, and transformed cells were induced with 0.5 mM IPTG. The expression profile before and after induction shows the presence of an approximately 32-kDa protein in the clarified lysate of cells transformed with pPGgp47 that is absent in the lysate of cells transformed with pET28a vector alone ( Figure 5A). However, most of the induced protein localizes in the insoluble fraction, and only a small proportion of the protein is soluble. Because renaturation of protein recovered from the insoluble fraction was unsuccessful, gp47 was partially purified (˜75% homogeneity) from the soluble fraction by passage through a Ni-NTA column ( Figure 5A).

RDFs typically inhibit integrative recombination as well as stimulate excisive recombination in vitro (e.g., λ [ 7], P2 [ 46], φRv1 [ 36]); since the substrates and reaction requirements for excision were not yet established, we first explored the ability of Bxb1 gp47 to inhibit integrative recombination. We have previously described an in vitro integrative recombination assay in which one of the partner DNAs is part of a supercoiled plasmid and the other is a short linear DNA [ 34]. Addition of purified gpInt catalyzes formation of a linear DNA product containing *attL* and *attR* that can be readily separated from both substrates by agarose gel electrophoresis. Addition of Bxb1 gp47 was observed to strongly inhibit integrative recombination, whereas a similar protein preparation extracted from control cells without Bxb1 gp47 did not ( Figure 5B). Bxb1 gp47 is thus a potent inhibitor of Bxb1 integrative recombination.

Tyrosine recombinases utilize relatively large *attP* substrates with multiple binding sites for integrase and accessory factors leading to the formation of complex architectures at these sites—their cognate RDFs function by influencing the formation or disruption of these nucleoprotein architectures at specific substrates. Although Bxb1 integration requires only short DNA substrates (38 bp in *attB* and 48 bp in *attP*) and does not require accessory proteins, we initially used relatively large (>300 bp) segments of *attL* and *attR* DNA to establish an in vitro excisive recombination assay in case either auxiliary Int-binding sites or Xis-binding sites are required for excision, as in the tyrosine integrase systems. Addition of purified gpInt and gp47 to a reaction in which one DNA is on a supercoiled plasmid and the other a short linear DNA results in the formation of a linear product, the amount of which increases with increasing concentration of gp47 ( Figure 5C, panel a). No recombinant product is obtained when gpInt and a control extract is added (panel b); recombination also does not occur when only gp47 or gpInt is present or when only one of the recombining DNAs is present in the assay ( Figure 5C, panel c).

In order to determine the site requirements for excision, in vitro recombination was performed using large (˜350 bp) *attL* or *attR* DNA in supercoiled plasmids and varying sizes of linear partner DNAs. Recombination takes place efficiently in vitro even when short (50 bp) partner DNA is used ( Figure 5D); the efficiency of the reaction remains unchanged even when *attL* and *attR* are both less than 60 bp ( Figure S2) demonstrating that like integration, excisive recombination uses short substrate DNA sequences (≤50 bp) and there is no requirement for accessory Int-binding sites in *attL* and *attR* for excisive recombination. DNase I footprinting confirms that gpInt protects a region of approximately 50 bp surrounding the cleavage site in both *attL* and *attR* ( Figure S3). Since these substrates are too small to include additional Xis-binding sites, Bxb1 gp47 presumably either binds DNA within the core Int-binding region, or alternatively may not interact directly with DNA at all and influences the direction of the reaction solely through protein–protein interactions with gpInt.

### DNA-Binding Properties of gp47

**Figure 6:**
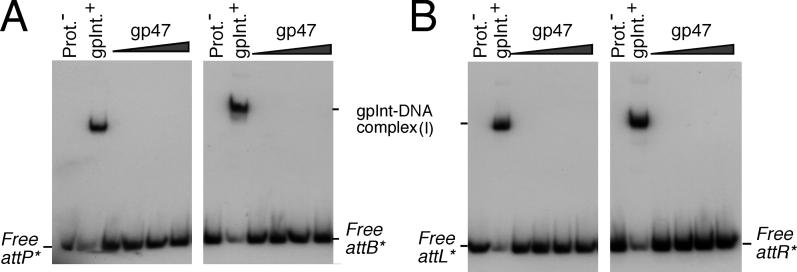
. DNA-Binding Properties of Bxb1 gp47 (A and B) The ability of gp47 to bind *attP, attB, attL,* and *attR* was determined using native gel electrophoresis. Binding of gp47 or gpInt to *attP* and *attB* (A) or *attL* and *attR* (B) was performed with either gpInt (0.072 μM), or increasing concentrations of gp47 (0.45, 0.89, 1.78, and 3.56 μM).

The ability of gp47 to bind *attP, attB, attL,* and *attR* DNA was examined using native gel electrophoresis ( Figure 6). Although an approximately 50-bp segment of all four DNAs is sufficient for integration and excision [ 37] we initially used larger substrates (˜350 bp) to study gp47 binding. Interestingly, gp47 does not show any detectable binding to any of the four *att* DNAs at any protein concentration using native gel electrophoresis ( Figure 6A and 6B), and we have been unable to detect the binding of gp47 to *att* DNA using filter-binding assays (unpublished data). Thus, in contrast to all other characterized RDFs, Bxb1 gp47 does not have the ability to bind DNA.

**Figure 7:**
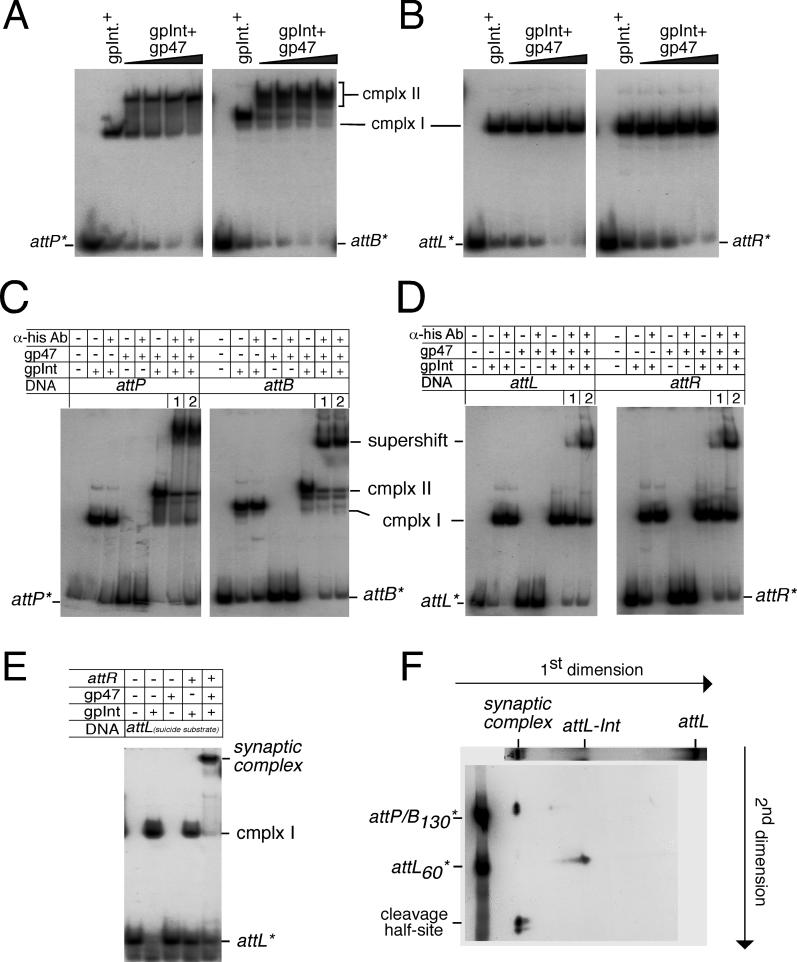
Substrate-Dependent Interaction of Bxb1 gpInt and gp47 (A and B) Binding of gpInt and gp47 to *attP*/ *attB*/ *attL*/ *attR* was monitored by native gel electrophoresis. Radiolabeled DNA fragments (˜300 bp) were incubated with either gpInt (0.072 μM) alone or gpInt with increasing concentrations of gp47 (0.45, 0.89, 1.78, and 3.56 μM). The positions of DNA–gpInt complexes (cmplx I) as well as tertiary complexes containing DNA, gpInt, and gp47 (cmplx II) are shown. (C and D) The presence of gp47 in the tertiary complexes shown in (A) was determined by the ability of α-His antibodies to supershift complexes observed by native gel electrophoresis. α-His antibodies were either added to reactions containing DNA, gpInt, and gp47 (indicated as lane 1), or first preincubated with gp47 for 30 min and then added to reactions containing DNA and gpInt (lane 2). The protein–DNA complexes were separated from free DNA on a 5% native PAGE. The positions of the tertiary complexes of gp47, gpInt, and DNA as well as the antibody supershifted complexes are indicated. (E and F) Bxb1 gp47 is required for trapping a synaptic complex in excision. A suicide substrate version of *attL* DNA (5′ radiolabeled at both ends) was used that has a nick on the top strand positioned four bases to the 5′ side of the scissile bond. Bxb1 gpInt (72 nM) binds normally to this substrate to form Complex I (cmplx I), but when *attR* partner DNA (200 bp) and gp47 (3.56 μM) is added, no recombinant products are released. Instead, a prominent slow-moving complex is observed that absolutely requires Bxb1 gp47 for its formation. We have identified this as a synaptic complex using 2D-PAGE (F). In brief, a vertical gel slice was removed from the last lane in panel E, incubated with proteinase K and SDS, and then electrophoresed through a second dimension. Approximately 50% of the radiolabeled DNAs in this complex correspond to *attP* recombinant product and 50% correspond to a cleaved half-site. The bottom of the gel slice containing unbound *attL* DNA was removed prior to the second dimension of electrophoresis. Further details on the characterization of these suicide substrates will be described in future publications.

Since Bxb1 gp47 does not bind DNA directly, we examined whether it is able to alter Int–DNA complexes, presumably via protein–protein interactions ( Figure 7). We have shown previously that Bxb1 gpInt forms a single well-defined protein–DNA complex (Complex I) with each of the four *att* DNAs [ 37] ( Figure 6). However, when gp47 is included in binding reactions with either *attP* or *attB,* an additional slower migrating complex (Complex II) is observed ( Figure 7A). The presence of gp47 in Complex II was confirmed by the ability of α-His antibodies (recognizing the N-terminal hexahistidine tag of gp47) to supershift Complex II formed with *attP* or *attB* ( Figure 7C). Association of gp47 with Int– *att* complexes could conceivably be mediated by either protein–protein interactions or by Int-dependent binding of gp47 to DNA, but the observation that Complex II forms with a smaller *attB* substrate (60 bp)—most of which is involved in interactions with gpInt ( Figure S4)—argues against the latter possibility. We therefore favor the idea that gp47 interacts with gpInt– *attP* and gpInt– *attB* complexes through protein–protein interactions with gpInt, although interactions between gp47 and the DNA cannot be entirely ruled out (and we note that the 8 bp at the centers of all four sites are identical). Thus, in contrast to the architectural roles of Xis in the tyrosine integrase systems, gp47 presumably acts to inhibit integrative recombination by either stearically blocking synapsis of gpInt– *attP* and gpInt– *attB* complexes, or by changing the conformation of these complexes so that they either no longer synapse or synapse but cannot undergo strand exchange.

Curiously, supershifted complexes are not formed when gpInt and gp47 are added to approximately 350-bp *attL* or *attR* DNA substrates ( Figure 7B), although they are weakly formed with high concentrations of gp47 with shorter (60 bp) DNA substrates ( Figure S4). To address the trivial explanation that the reaction conditions are simply not permissible for excision (or Complex II formation), we performed similar reactions containing both *attL* and *attR* DNAs (unpublished data) and showed that recombination does indeed occur. Thus, under recombination-permissive conditions, gp47 associates only weakly or unstably with gpInt– *attL* and gpInt– *attR* complexes, in sharp contrast to its more stable association with *attP* and *attB* complexes. The nature of the gp47–gpInt interaction is thus dependent on the specific DNA site to which gpInt is bound.

Interestingly, addition of α-His antiserum to reactions containing gpInt, gp47, and either *attL* or *attR* DNA generates a supershifted complex ( Figure 7D) consistent with the formation of weak or unstable complexes that can be stabilized by antibody binding. To exclude the possibility that a tertiary stable complex does form with *attL* and *attR,* but migrates with the same mobility as Complex I ( Figure 7), we have performed binding experiments with an MBP-gp47 fusion protein (unpublished data). Although this fusion protein does not support excision, it forms Complex II with both *attP* and *attB* DNAs that migrate considerable slower than with native gp47. Nevertheless, no Complex II is seen with MBP-gp47 and either *attL* or *attR* DNA (unpublished data). Finally, the order of addition of gp47 and the anti-His antibodies makes a significant difference to the amount of the supershifted complex ( Figure 7D) with *attL* and *attR,* in a manner that is not seen with *attP* or *attB* ( Figure 7C). We conclude from these experiments that Bxb1 gp47 binds weakly to gpInt– *attL/R* complexes and that the bound forms are unstable during gel electrophoresis.

### A Model for the Role of Bxb1 gp47 in the Control of Recombination Directionality

Bxb1 gp47 is an unusual RDF and modulates the directionality of integrase-mediated site-specific recombination by a mechanism that is quite distinct from that used by the tyrosine integrase systems. The data presented here show that gp47 does not act through direct DNA interactions, but by changing the ability of gpInt–DNA complexes to participate further in the reaction pathway. The mechanism for the inhibition of integrative recombination may be relatively simple, involving the association of gp47 with gpInt– *attP* and Int– *attB* complexes—via protein–protein interactions—and preventing them from productively associating. However, it is noteworthy that these proposed gp47–gpInt interactions are qualitatively different, depending on the DNA substrate to which gpInt is bound. This is consistent with a model we have proposed previously [ 39] suggesting that the conformations of gpInt–DNA complexes are different depending on the specific DNA substrate that is bound, and that the compatibility of the various possible conformations plays a role in the selection of sites for productive synapsis. It is, however, interesting that gp47 associates with both *attP*- and *attB*-bound complexes since these two substrates are quite distinct in both their sequence and in their length requirements and likely adopt different conformations [ 37, 39].

How does Bxb1 gp47 promote excisive recombination without stably associating with gpInt– *attL/R* complexes? There are three main possibilities. First, gp47 could enzymatically modify gpInt in a manner that confers the ability to perform excisive recombination. However, this explanation seems unlikely since titration experiments show that gp47 acts stoichiometrically and not enzymatically ( Figure 5C, unpublished data). Second, gp47 could act by shifting the dynamic equilibrium between a series of reversible reactions that usually favor integration. Thus, by binding to gpInt– *attB* and – *attP* complexes and preventing them from participating further in these reactions, *attP* and *attB* would eventually accumulate as products. Third, gp47 could associate with gpInt– *attL* and gpInt– *attR* complexes—albeit with lower affinity and perhaps unstably, and stimulate either synapsis of *attL* and *attR* or the initiation of strand exchange. To distinguish between these latter two models, we have utilized a suicide *attL* substrate (similar to those described for the lambda system [ 47, 48]) that promotes the accumulation of synaptic complexes containing cleaved substrates covalently joined to gpInt ( Figure 7E and 7F). Formation of these complexes is strongly dependent on the presence of gp47 ( Figure 7E), eliminating the simple equilibrium model and strongly supporting a role for gp47 generating productive synaptic complexes.

### Concluding Remarks

We have demonstrated here that mycobacteriophage Bxb1 utilizes a novel RDF to regulate the directionality of recombination by its serine integrase. It is relatively large, does not bind to DNA, does not act by the remodeling of macromolecular architectures, and associates with Bxb1 gpInt in a DNA-dependent manner. Although it is not known what other functions gp47 might possess, its presence in other phages that use different integration systems, and its gene location within other replication functions, suggest that it may also act in DNA replication. While some recombination accessory proteins—including the RDFs of phages P2 and HP1 (Cox)—have dual functions [ 23, 24], all of these act through DNA binding, so that although the biological roles may be quite distinct, the mechanisms are based on common DNA-binding specificities. Since Bxb1 gp47 appears to associate with gpInt in a highly specific manner that is dependent on the particular DNA substrate bound, it raises the interesting question as to whether it performs its other activities through similar and highly specific interaction with other phage-encoded—or possibly host-encoded—proteins in unrelated processes such as DNA replication.

## Materials and Methods

### Bacterial strains and growth media.

M. smegmatis mc ^2^155 was grown in Middlebrook 7H9 or 7H10 (Difco, Sparks, Maryland, United States) medium supplemented with ADC and the appropriate antibiotics; the liquid 7H9 medium in addition contained 0.05% Tween 80. Kanamycin, hygromycin, and sucrose were used at concentrations of 40 μg/ml, 150 μg/ml, and 10% w/v, respectively.

### Construction of Bxb1 genomic DNA library.

Bxb1 phage DNA was prepared, sheared, and a library constructed as described previously [ 42]. In brief, approximately 10 μg of DNA was hydrodynamically sheared, repaired, and size fractionated on an agarose gel; approximately 3-kb fragments were purified and ligated to the Hpa1 site of plasmid vector pPG1. Plasmid pPG1 is a derivative of pJL37 [ 43] in which the phsp60 region has been removed using Nde1 and Xba1 followed by religation; it confers kanamycin resistance and replicates both in *E. coli* and M. smegmatis. Approximately 200 colonies obtained were pooled, and DNA was prepared using a Qiagen Midi prep protocol (Qiagen, Valencia, California, United States) and then analyzed by restriction digestion to confirm the heterogeneity of the cloned fragments.

### Construction of excision tester strains.

The Cla1-Dra1 fragment of pSD26 containing the hygromycin-resistance cassette was cloned into the Dra1 site of pSJ25b [ 34] (containing 29,081–31,030 bp of the Bxb1 genome encoding *attP* and gpInt); the resultant plasmid is pSJ25Hyg. The SacB cassette was amplified by PCR from pAVN30 (kind gift from Dr. William Jacobs) and cloned into the Xmn1 site of pSJ25Hyg to obtain the integrative plasmid pPGA1. Plasmid pPGA1 was transformed into M. smegmatis mc ^2^155 to produce the “excision tester strain” in which the *int, hyg,* and *sacB* genes are flanked by *attL* and *attR;* the correct integration of pPGA1 into *attB* was confirmed by PCR.

A derivative of pPGA1 lacking *int* (pPGA2) was integrated into the *attB* site of mc ^2^155 by co-electroporation with a plasmid providing transient expression of *int* and which lacks oriM. This generated a *hyg ^R^ sac ^S^ int* ^−^ excision tester strain.

### Construction of plasmids.

A plasmid recovered from the genomic DNA library that exhibited excision activity contains phage DNA from coordinates 35,190–37,500 bp; this was designated as pPGX1 and contains Bxb1 genes *49*– *45*. Derivatives of pPGX1, designated pPGX2–6 (with different orientations of insert relative to vector background designated as a and b) and containing truncations from both ends, were generated by PCR and cloned into the Hpa1 site of pPG1. Further truncations were made in pPGX6b, and the plasmids were designated as pX6b1–5. A plasmid for overexpression of Bxb1 gp47 was constructed by PCR amplification of gene *47,* digestion of the product with Nde1 and Xho1, and cloning into the Nde1-Xho1 site of pET28a (Novagen, Madison, Wisconsin, United States). The resulting plasmid (pPGgp47) expresses Bxb1 gp47 with a His _6_ tag at the N-terminus.

### In vivo assay for site-specific excisive recombination of Bxb1.

The excision tester strain was transformed with either a Bxb1 DNA library or specific plasmids and equal amounts of the transformation mixture plated on 7H10 plates containing kanamycin (40 μg/ml), kanamycin (40 μg/ml) and hygromycin (150 μg/ml), and kanamycin (40 μg/ml) and 10% sucrose.

### Protein purification of Bxb1 Integrase and gp47.

Plasmids pET28a and pPGgp47 were transformed into BL21 (DE3) pLysS (Novagen) and grown in Luria Bertani (LB) broth to an optical density (OD) of 0.6 at 30 °C and induced for 4 h at 22 °C with 0.6 mM IPTG. The cells were lysed in a buffer containing 50 mM Tris-HCl (pH 8.0), 300 mM NaCl, 5% glycerol, and 0.1 mM DTT, and the clarified supernatants were loaded onto Ni-NTA columns (Qiagen). The columns were washed with 30 mM and 50 mM imidazole, and eluted with 150 mM imidazole.

A C-terminally His-tagged version of Bxb1 gpInt [ 34] was purified following overexpression of the protein from pInt-His in E. coli BL21 pLysS, followed by nickel-affinity chromatography (Qiagen) as described [ 34]. Native gpInt lacking a His tag was overexpressed from pMA1 and purified using heparin-agarose affinity chromatography [ 34].

### In vitro recombination assay.

In vitro integrative recombination was carried out between *attP* in pAIK6 and linear *attB* in a recombination buffer containing 20 mM Tris-HCl (pH 7.5), 10 mM EDTA, 25 mM NaCl, 10 mM spermidine, and 1 mM DTT with or without the addition of purified protein (either from the empty vector control or pPGgp47), and the products were separated on a 0.8% agarose gel and visualized by ethidium bromide staining.

In vitro excisive recombination was carried out between 300 bp of *attL* in pMOS- *attL* and linear *attR* of indicated sizes in the above recombination buffer; gpInt and gp47 were added as indicated. The reactions were carried out at 25 °C for 2 h and separated on a 0.8% agarose gel.

### DNA-binding assays.

Approximately 5 ng of labeled DNA was incubated with the indicated amounts of either intact gpInt (His tagged or native) and/or gp47 in a buffer containing 20 mM Tris-HCl (pH 7.5), 10 mM EDTA, 25 mM NaCl, 10 mM spermidine, 1mM DTT, and 1 μg calf thymus DNA, in a total volume of 10 μl and incubated at 25 °C for 1 h. Where indicated, α-His antibodies were added to a final concentration of 60 μg/μl. Antibody supershifts were performed either by preincubating gp47 with the antibodies for 30 min followed by addition to a reaction containing DNA and gpInt or by the addition of the antibody to a premixed (30 min) sample containing DNA, gpInt, and gp47 followed by a further incubation of 30 min. Protein–DNA complexes were separated from free DNA on a 5% non-denaturing polyacrylamide gel at 10 °C.

### Suicide substrates and analysis of synaptic complexes.

A suicide substrate derivative of *attL* was generated by annealing three oligonucleotides: a 60-base bottom strand oligonucleotide, and 27-base and 33-base top strand oligonucleotides. Prior to annealing, the 33-base oligonucleotide was phosphorylated with cold ATP, and both the 60-base and 27-base oligonucleotides were radiolabeled with ^32^P at their 5′ ends using polynucleotide kinase. Addition of gpInt, gp47, and unlabeled *attR* DNA (200 bp) generates a slow-migrating synaptic complex. For 2D-PAGE analysis, a vertical gel slice was removed and incubated in buffer containing 1 mg/ml proteinase K for 10 min followed by addition of 0.5% SDS for an additional 10 min. The gel slice was place across the top of a 10% polyacrylamide gel containing 0.05% SDS, and electrophoresed in the second dimension.

## Supporting Information

Figure S1 Identification of Bxb1 RDF (356 KB TIF)

Figure S2 In Vitro Excisive Recombination Using Short *attL* and *attR* Substrates
(1.0 MB TIF)

Figure S3 DNaseI Footprinting of gpInt–DNA Complexes (5.4 MB TIF)

Figure S4 Binding of gpInt and gp47 to Short DNA Substrates (2.8 MB TIF)

Table S1 Mutagenesis of pPGX6b (42 KB PDF)

## Abbreviations

aa: amino acid
bp: base pair
ORF: open reading frame
RDF: recombination directionality factor
