# Supplementary material for: Control of Phage Bxb1 Excision by a Novel Recombination Directionality Factor
Source: PLoS Biol. 2006 May 30;4(6):e186. doi: 10.1371/journal.pbio.0040186 (PMC1470463; doi:10.1371/journal.pbio.0040186)
Supplement: Supplementary file 1 [file pbio.0040186.st001.pdf]

Table S1

Co-ordinates Of Mutations	Base Change	Corresponding mutations in:						Residue mutated in gp47
		Frame 1	Frame 2	Frame 3	Frame 4	Frame 5	Frame 6	
36724	C-A	Q-K	C-*	A-E	A-S	C-F	L-F	22
36694	C-T	Q-*	**S-S**	P-L	G-R	W-*	**L-L**	32
36537	G-A	G-D	A-T	**R-R**	**S-S**	P-S	A-V	84
36533	G-A	W-*	G-E	D-N	S-F	**I-I**	V-S	85
36430	C-T	L-F	**C-C**	A-V	A-T	S-N	**E-E**	120
36354	T-G	G-E	G-S	**R-R**	**A-A**	P-S	P-L	145
36331	C-T	S-A	Y-*	I-S	I-L	D-A	**G-G**	153
36328	G-A	Q-*	**P-P**	P-L	G-R	W-*	**L-L**	154
36298	C-T	A-T	**V-V**	C-Y	H-Y	A-V	**G-G**	164
36297	C-T	A-V	P-S	Q-C	W-*	A-T	G-D	164
36279	A-G	S-F	P-S	**V-V**	**R-R**	D-N	G-E	170
36262	G-A	T-A	**T-T**	H-R	C-R	V-A	**R-R**	176
36250	G-A	A-T	**R-R**	G-D	P-S	A-V	**S-S**	180
36244	G-C	V-L	**S-S**	R-P	**R-R**	T-R	D-E	182
36226	T-C	Y-H	**G-G**	V-A	T-A	Y-C	**V-V**	188
36193	A-G	M-V	**A-A**	H-R	C-R	M-T	**H-H**	199
36190	G-A	E-K	W-*	G-E	P-L	S-F	**F-F**	200
36128	G-A	W-*	G-D	A-T	A-V	**C-C**	P-S	220
36081	C-T	A-V	**L-L**	**G-G**	**Q-Q**	A-T	S-N	236
36037	C-T	Q-*	**A-A**	P-L	G-S	W-*	L-L	251
